# PERSON-CENTERED CARE WHEN OLDER ADULTS WITH DEMENTIA AND THEIR FAMILY CAREGIVERS HOLD DIFFERENT CARE PRIORITIES

**DOI:** 10.1093/geroni/igad104.2886

**Published:** 2023-12-21

**Authors:** Emma Quach, Martina Azar, Katherine Kennedy, Catherine Dawson, Chelsea Hawley

**Affiliations:** VA Bedford Healthcare System, Bedford, Massachusetts, United States; VA Boston Health Care, Boston, Massachusetts, United States; Providence VA Medical Center, Providence, Rhode Island, United States; VA Bedford Healthcare System, Bedford, Massachusetts, United States; VA Bedford Healthcare System, Bedford, Massachusetts, United States

## Abstract

In person-centered care, clinicians develop care plans that incorporate “what matters most” to patients (i.e., their priorities, concerns, and preferences), a process complicated when patients’ family caregivers have their unique priorities. To investigate care planning with patients with dementia and their family caregivers, we audio-recorded, transcribed, and thematically analyzed 4 medical visits dually attended in 2022 by older patients with dementia and their spousal/adult child caregiver(s). In these visits clinicians asked both about their priorities/concerns, which mostly complemented but sometimes conflicted with each other. Older adults’ priorities related mostly to social activities and minimally to health care, emphasizing continuity. Older adults strived to resume past social activities (farmer’s market), continue enjoyable life-long activities (sports), maintain non-problematic medication regimens, and retain the same home health nurse. Caregivers’ priorities focused on emergent physical problems (patient’s worsening memory and leg pain), service access, and care coordination. When older adult and caregiver priorities did not conflict, clinicians incorporated priorities from both sources into care plans (initiated referrals for farmer’s market). When older adults and caregivers’ priorities conflicted (exercise), clinicians simultaneously acknowledged family caregiver’s concerns, expressed their (clinician) views, honored older adults’ preferences, and invited them to re-assess such preferences in future medical visits. By asking “what matters most” to both patients and their family caregivers, clinicians gain rich insights about both parties to guide care planning. Future research should examine care planning with more dementia dyads to identify care planning practices that promote both patient and caregiver outcomes and ultimately enable aging in place.

